# Degree of Left Renal Vein Compression Predicts Nutcracker Syndrome

**DOI:** 10.3390/jcm7050107

**Published:** 2018-05-08

**Authors:** Patrick T. Hangge, Nikhil Gupta, Aditya Khurana, Keith B. Quencer, Hassan Albadawi, Sadeer J. Alzubaidi, M-Grace Knuttinen, Sailendra G. Naidu, Rahmi Oklu

**Affiliations:** 1 Department of General Surgery, Mayo Clinic, Phoenix, AZ 85054, USA; hangge.patrick@mayo.edu; 2 Division of Vascular and Interventional Radiology, Minimally Invasive Therapeutics Laboratory, Mayo Clinic, Phoenix, AZ 85054, USA; n3gupta@gmail.com (N.G.); khurana.aditya@mayo.edu (A.K.); kbquencer@gmail.com (K.B.Q.); albadawi.hassan@mayo.edu (H.A.); Alzubaidi.Sadeer@mayo.edu (S.J.A.); Knuttinen.Grace@mayo.edu (M.-G.K.); naidu.sailen@mayo.edu (S.G.N.); 3 Tufts University School of Medicine, Boston, MA 02111, USA; 4 Mayo Clinic School of Medicine, Mayo Clinic, Scottsdale, AZ 85259, USA; 5 Division of Interventional Radiology, Department of Radiology, University of Utah, Salt Lake City, UT 84112, USA

**Keywords:** nutcracker syndrome, left renal vein, compression

## Abstract

Nutcracker syndrome (NS) refers to symptomatic compression of the left renal vein (LRV) between the abdominal aorta and superior mesenteric artery with potential symptoms including hematuria, proteinuria, left flank pain, and renal venous hypertension. No consensus diagnostic criteria exist to guide endovascular treatment. We aimed to evaluate the specificity of LRV compression to NS symptoms through a retrospective study including 33 NS and 103 control patients. The size of the patent lumen at point of compression and normal portions of the LRV were measured for all patients. Multiple logistic regression analyses (MLR) assessing impact of compression, body mass index (BMI), age, and gender on the likelihood of each symptom with NS were obtained. NS patients presented most commonly with abdominal pain (72.7%), followed by hematuria (57.6%), proteinuria (39.4%), and left flank pain (30.3%). These symptoms were more commonly seen than in the control group at 10.6, 11.7, 6.8, and 1.9%, respectively. The degree of LRV compression for NS was 74.5% and 25.2% for controls (*p* < 0.0001). Higher compression led to more hematuria (*p* < 0.0013), abdominal pain (*p* < 0.006), and more proteinuria (*p* < 0.002). Furthermore, the average BMI of NS patients was 21.4 and 27.2 for controls (*p* < 0.001) and a low BMI led to more abdominal pain (*p* < 0.005). These results demonstrate a strong correlation between the degree of LRV compression on imaging in diagnosing NS.

## 1. Introduction

Nutcracker syndrome refers to symptomatic compression of the left renal vein (LRV) by the aorta and superior mesenteric artery (SMA). It was first described in 1950 by El-Sadr and Mina and was named in 1971 [1,2,3,4]. Differing views on definitive diagnostic criteria and varied symptomatology have led to sparse epidemiological data in the literature [3]. Early reports suggested that this condition was rare. However, increasing numbers of patients are diagnosed after failing treatment for nephropathies [1]. The true prevalence in the general population is unknown.

Anatomic nutcracker features may exist without sequelae (referred to as left venous renal entrapment syndrome or ‘nutcracker phenomenon’) [5]. One study found over 50% narrowing of the left renal vein in 27% of CT angiograms in healthy adults [6]. However, ‘nutcracker syndrome’ (NS) is characterized by coexisting of the nutcracker anatomy and characteristic symptoms. As it is a rare disorder, many other diseases are included in the workup including urinary tract infections, nephropathy, idiopathic hypercalciuria, and urolithiasis [3,7]. Symptoms of NS may include most commonly abdominal pain, left flank pain, and macroscopic hematuria [7]. Microscopic hematuria, proteinuria, left-sided varicocele (male), pelvic congestion syndrome, dysmenorrhea, and dyspareunia (female) have also been observed [1,3,8]. NS most commonly presents in the second and third decade. There is a female preponderance in the literature, especially during pregnancy [1,3]. A review of the literature by Rudloff et al., found 62/80 (78%) NS patients to be female [9]. NS is also more commonly seen in patients with lordosis or history of rapid vertical growth [1]. The causative nature of LRV compression and symptoms is fairly controversial. Studies have been unable to distinguish symptoms that can be directly attributed to the compression of the LRV; however this theory is the most common understanding of this disease etiology.

**Figure 1:**
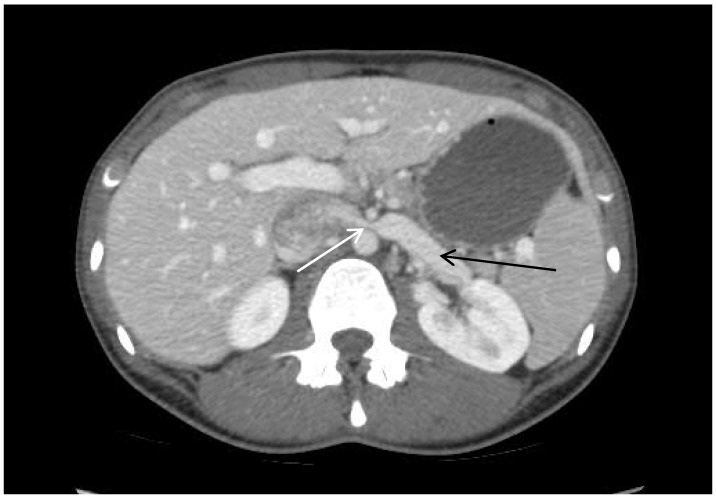
Nutcracker anatomy on computed tomography. White arrow points to compression of the left renal vein (LRV) between the superior mesenteric artery (SMA) and aorta. The black arrow points to LRV dilation.

The compression of the LRV can occur primarily in two anatomic locations. Anterior NS occurs at the branching of the SMA off of the aorta (Figure 1). Posterior NS, a rarer form, exists when a retroaortic LRV is compressed between the aorta and vertebrae. These compressions can often lead to the distal distension of the renal and gonadal veins as well as increased venous blood pressure. This can be exacerbated by orthostatic hemodynamic changes, leading to more severe proteinuria and hematuria [1,3]. Frequently, collateral vessels form to reduce pressure.

The diagnosis of NS involves a combination of clinical history, laboratory studies, and radiological investigation. History and physical exam is the most useful in initial assessment. The compression of the LRV can be determined via Doppler ultrasonography, renal vein angiography (with possible adjunctive intravascular ultrasound and/or pressure measurements), computed tomography (CT), and magnetic resonance imaging [1,3,10]. Intravenous urograms and retrograde pyelograms are not sensitive [3]. No definitive radiologic diagnostic criteria exist and subjective interpretation of images is used to make the imaging diagnosis. We aim to evaluate the association and degree of LRV compression to symptoms to help provide more objective imaging criteria in the diagnosis of NS.

## 2. Materials and Methods

This retrospective study was approved by our Institutional Review Board with a waiver of the need for informed consent. The authors have a combined 40 years of experience treating venous congestion disorders including nutcracker syndrome. Our institution is a major referral center and this syndrome is commonly seen. We utilized Illuminate (Softek) Software (Overland Park, KS, USA) to perform a search of our institution’s radiology electronic database for the terms ‘nutcracker’ and ‘nutcracker syndrome’ for all patients above the age of 20 who had either computed tomography angiography (CTA) or computed tomography venography (CTV). Standard CT arteriogram and venogram protocols were followed. The search was restricted to patients examined for a period of 13 years from 2003 to 2016 and identified patients who presented with nutcracker anatomy on computed tomography. The electronic medical records of each patient were reviewed for clinical notes indicating nutcracker syndrome diagnosis. 42 patients were initially identified with nutcracker anatomy suspicious for NS by cross-sectional imaging. Diagnosis was confirmed in a total of 33 patients with NS. The control group included 103 sequential abdominal computed tomography (CT) studies with varying medical conditions to reduce selection bias. Patient medical records were further reviewed to record specific symptoms or signs the patient had related to nutcracker syndrome including hematuria, proteinuria, abdominal pain, left flank pain, and body mass index (BMI). A control study population was thereafter reviewed. The controls were also selected using Softek Illuminate by carrying out a search for patients who underwent CTA or CTV during the corresponding dates. In keeping with the analysis conducted for the NS patients, the medical records of the control patients were also reviewed for the corresponding clinical characteristics.

### 2.1. Image Analysis, Patient Presentation

CT measurements were made from axial images using a clinical image viewer and picture archiving system. Digitally calibrated measurement tools allowed for the measurement of the width of the LRV lumen, which was used to calculate the degree of compression. Two lumen measurements were recorded: (1) *compressed segment*—the diameter of the LRV at the point of maximal compression as the LRV passes between the SMA and aorta, and (2) *pre-compressed segment*—the diameter of the uncompressed segment of the LRV proximal to the passing between the aorta and SMA. The degree of compression was calculated by dividing the change in compression (*pre-compressed segment* value—*compressed segment* value) by the pre-compression value. A compression ratio (*CR*), representing the degree of venous compression, was calculated as the pre-compressed segment by the compressed segment:

$$CR=\;\frac{pre-compressed\;segment}{compressed\;segment}$$

These calculations were carried out in accordance with an analogous method of measuring iliac vein compression [11,12]. The BMI and clinical presentation of symptoms closest to the imaging date for each patient were recorded.

### 2.2. Statistical Analysis

A Student’s *t*-test was carried out to assess whether the difference in degree of venous compression between the groups was significant. A *p*-value of less than 0.05 was used as the cut-off for statistical significance. Multinomial logistic regression was used to identify the association between the extent of LRV compression and clinical symptoms, BMI, age, and other patient characteristics using software.

## 3. Results

### 3.1. Patient Characteristics and Symptoms

A total of 33 patients were diagnosed with nutcracker syndrome. The majority of patients were female (97.0%). The mean age was 46.2 ± 3.3 years (range 20–82 years) and average BMI was 21.4 ± 0.7. The control group comprised 103 patients. The majority were female (58.2%) and had a mean age 46.7 ± 1.7 years (range 20–80 years). Mean BMI was significantly higher than NS patients at 27.2 ± 0.5 (*p* < 0.0001). A majority of NS patients presented with abdominal pain (72.7%), followed by hematuria (57.6%), proteinuria (39.4%), and left flank pain (30.3%). The control patients presented significantly less frequently with each of the above symptoms (Table 1).

### 3.2. Imaging Characteristics

The degree of compression of the LRV was significantly greater for NS, 74.5 ± 1.9%, when compared to controls, 25.4 ± 2.4% (*p* < 0.0001). LRV diameters were similar at the site of pre-compression between NS and control groups, however significantly more narrow in NS patients at the site of compression. The ratio of pre-compression to the compression area (or analogous area of compression for controls) was 4.6 ± 0.3 for NS and 1.6 ± 0.1 for controls (*p* < 0.0001) (Table 2).

**Figure 2:**
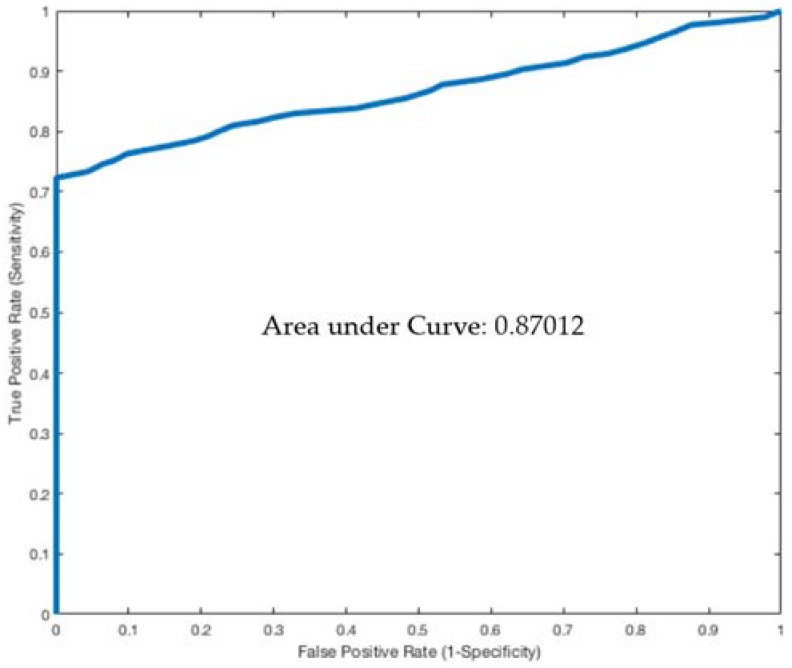
ROC curve for left renal vein ratio: pre-compressed to compressed.

Plotting the ratio of diameters at pre-compression to compression on a receiver operating characteristic (ROC) curve gives an area under the curve of 0.87 (Figure 2). Using a cutoff ratio of over 2.25 has 91% sensitivity and specificity for NS diagnosis. A cutoff of over 3.0 has 85% sensitivity and 100% specificity for NS.

MLR showed that higher compression was associated with hematuria, abdominal pain, and proteinuria. Furthermore, lower BMI led to more presentations of abdominal pain (*p* = 0.005). No variable predicted left flank pain with statistical significance. Age and gender were not predictors of these findings or symptoms (Table 3).

## 4. Discussion

Limited data and a lack of universal diagnostic criteria make the diagnosis of NS challenging. This can be problematic as patients with NS may require invasive treatment such as endovascular stenting or renal vein re-implantation. As such, this study sought to investigate whether greater LRV compression visualized on CT can be used as an indicator for NS with confidence and also investigate the relationship between the degree of LRV compression and patient symptoms. The results revealed that the degree of compression of the LRV in NS was significantly higher compared to controls (Table 2). A pre-compression to compression ratio of the LRV over 2.25 demonstrated 91% specificity and sensitivity for NS. This is consistent with the current evidence in the literature which report that the presence of a ‘beak sign’ or narrowing in the LRV between SMA and aorta with a proximal LRV dilation has a sensitivity of 91.7% and specificity of 88.9% for substantiating NS diagnosis [13,14]. It was also reported that a compression ratio greater than 4.9 at the hilar and aortomesenteric regions, which corresponds to the anatomical location where our study has based its compression measurements on, has a sensitivity of 66.7% and specificity of 100% for NS. A compression ratio of the LRV over 2.25 should raise suspicion for NS in the context of clinical symptoms. However, in the presence of compressed LRV alone, it is reasonable to observe the patient until symptomatic with severe, persistent abdominal pain, hematuria, proteinuria, or renal insufficiency.

The results of this study showed higher compression was associated with hematuria, abdominal pain, and proteinuria. Though the underlying pathophysiology of NS is not completely understood, it is believed that increased outflow restriction through abnormal aorto-SMA angle or branching elevates LRV pressure. NS is generally suspected when the pressure gradient between the LRV and inferior vena cava is above 2 mmHg compared to the normal gradient less than 1 mmHg [15]. Abdominal pain is believed to be caused by the inflammatory process triggered by LRV hypertension [16]. Hypertension is also suggested to cause fissure formation in thin veins through which blood enters the collecting system thereby generating hematuria and proteinuria [17,18]. Presumably, an increased compression should increase findings of these symptoms. It should be noted, however, that a distended LRV is not necessarily indicative of NS and it is estimated that approximately 51–72% of people exhibit distended LRVs [17]. It is suggested that the presence of hematuria is underutilized in diagnosing NS in children. Shin et al. examined a cohort of 216 children with isolated hematuria who underwent Doppler ultrasound examinations for NS. They reported that 33.3% of children with isolated hematuria had NS [19]. As 47% of isolated hematuria cases are not linked to a known cause, NS should perhaps be more frequently considered as a potential diagnosis [20].

Another physiological factor reported to be associated with NS is the lack of retroperitoneal fat which can reduce the aortomesenteric angle, causing LRV compression [21]. Lower BMI has been reported to be associated with higher incidence of NS and there is evidence suggesting that NS can develop post weight loss [22,23]. This is consistent with findings in this study in which NS patients were found to have a significantly lower BMI compared to normal controls. Additionally, patients with a low BMI were also more likely to present with abdominal pain. In general, patients with less total abdominal fat also have less visceral fat and this anatomical difference could potentiate abdominal pain in these patients. However, it is unclear why low BMI patients did not have an association with other symptoms such as hematuria, proteinuria, or flank pain. 

This study has several limitations. NS is an inherently rare disease and as such a retrospective study design was used to maximize the size of the patient cohort which was diagnosed with NS. Collectively, we were only able to find a small number of patients diagnosed with NS in our institution over the last 13 years. In addition, our control group possesses inherent bias in that they were selected from medical records of patients undergoing CT imaging for an abdominal complaint. Therefore, some intra-abdominal changes may be noted that would be absent in the general population. However, this effect was likely minimal. For future studies, it could be prudent to select the controls from potential kidney donors and as a consequence ensure a potentially healthy population undergoing a CT scan. None of the patients included in the study were diagnosed with posterior NS, rather we were only able to identify patient with the more common anterior NS.

In addition, our measurements were obtained using two-dimensional CT scans with single posture which may have limited our diagnosis precision. Differences in normal anatomy also make it challenging to define comprehensive criteria for NS. Other symptoms associated with NS which we did not include were varicoceles and orthostatic intolerance. We also did not evaluate LRV pressure as this data point was not recorded for all patients in the medical record.

## 5. Conclusions

This present study identifies a strong correlation between the degree of LRV compression on imaging in diagnosing NS. A pre-compression to compression ratio of the LRV over 2.25 demonstrated 91% specificity and sensitivity for NS. This study also identifies increased prevalence of hematuria, abdominal pain, and proteinuria in NS patients. Correlation with symptoms, laboratory results, and excluding other causes continues to be important in the workup of NS. Further large cohort and long-term follow-up studies will aid in the understanding of this rare disease. 

## Figures and Tables

**Table 1:** Baseline patient characteristics and symptom presentation.

	Nutcracker (*n* = 33)	Controls (*n* = 103)	*p*-Value
Female (%)	32 (97.0%)	60 (58.2%)	0.001
Age, years	46.2 ± 3.3	46.7 ± 1.7	0.5
BMI	21.4 ± 0.7	27.2 ± 0.5	<0.0001
Hematuria	19 (57.6%)	12 (11.7%)	<0.0001
Left Flank Pain	10 (30.3%)	2 (1.9%)	<0.0001
Abdominal Pain	24 (72.7%)	11 (10.6%)	<0.0001
Proteinuria	13 (39.4%)	7 (6.8%)	0.001

**Table 2:** Left renal vein compression imaging characteristics.

	Nutcracker	Controls	*p*-Value
Compression Percentage	74.5 ± 1.9	25.2 ± 2.4	<0.0001
Diameter Pre-Compression (mm)	10.7 ± 0.4	10.2 ± 0.4	0.42
Diameter at Compression (mm)	2.6 ± 0.2	7.0 ± 0.4	<0.0001
Ratio of Diameter at Compression vs. Pre-Compression	4.6 ± 0.3	1.6 ± 0.1	<0.0001

**Table 3:** Multi-logistic regression model for impact of age, gender, BMI, percent left renal vein compression on symptom and laboratory presentation.

	Hematuria		Proteinuria		Abdominal Pain		Left Flank Pain	
	Coeff (SD)	*p*-Val	Coeff (SD)	*p*-Val	Coeff (SD)	*p*-Val	Coeff (SD)	*p*-Val
Age	0.01 (0.01)	0.31	0.01 (0.01)	0.67	0.01 (0.01)	0.27	0.004 (0.02)	0.82
Gender	−0.64 (0.63)	0.31	−0.83 (0.83)	0.32	0.64 (0.60)	0.28	1.0 (1.1)	0.38
BMI	−0.07 (0.06)	0.23	0.07 (0.89)	0.34	−0.19 (0.07)	0.005	0.004 (0.09)	1.0
Percent LRV Compression	−0.02 (0.01)	0.01	−0.04 (0.01)	0.002	−0.03 (0.01)	0.007	0.03 (0.02)	0.06
